# Research Progress on Rhizosphere Microbiota for Controlling Soil-Borne Diseases: Mechanisms, Applications, and Challenges

**DOI:** 10.3390/microorganisms14040900

**Published:** 2026-04-16

**Authors:** Yong Liu, Xiaofang Sun, Jia Lai, Shugu Wei, Yuzhen Sheng, Yinchao Zhang, Qianfang Zhang, Pengsheng Ye, Ling Huang, Hualan Zeng

**Affiliations:** Industrial Crop Research Institute, Sichuan Academy of Agricultural Sciences/Horticultural Crops Germplasm Innovation and Utilization Key Laboratory of Sichuan Province, Chengdu 610300, China; liuyong918@126.com (Y.L.); sunxiaofang207@163.com (X.S.); laijia028@163.com (J.L.); weishugu@163.com (S.W.); shengyuzhen0516@126.com (Y.S.); yinchao1@126.com (Y.Z.); zqfzzf@126.com (Q.Z.); yeps18@163.com (P.Y.)

**Keywords:** rhizosphere microbes, soil-borne diseases, root exudates, biological control

## Abstract

Soil-borne diseases pose a severe threat to global agricultural production and food security. Traditional chemical control methods face significant challenges, including environmental pressure, pathogen resistance, and food safety concerns. The rhizosphere microbial community, often termed the plant’s ‘second genome’, plays a pivotal role in maintaining plant health and defending against pathogen invasion. Recent advances in multi-omics technologies, synthetic microbial communities (SynComs) construction, and rhizosphere metabolomics have significantly advanced our understanding of the mechanisms by which rhizosphere microbiomes suppress soil-borne diseases. This review systematically summarizes the following: 1. key drivers of rhizosphere microbial community assembly, particularly plant “cry for help” signaling; 2. core beneficial microbial taxa and their disease-suppressive mechanisms; 3. the critical role of microbial interaction networks; 4. microbiome-based management strategies and their application progress; and 5. current challenges and future research directions. Compared with previous reviews that separately discussed rhizosphere microbiota, disease-suppressive soils, synthetic microbial communities (SynComs), or prebiotics, this review uniquely integrates multiple levels of regulation, from plant genetic determinants (‘*M* genes’) and root exudate-mediated ‘crying for help’ to microbiome engineering (SynComs and prebiotics) and cross-kingdom interactions (bacteria–fungi–protists–phages). A central conceptual axis of ‘*M* genes → microbiome engineering → breeding’ is proposed, bridging plant genetics, microbial ecology, and crop improvement for durable disease suppression. Ultimately, this work aims to provide a theoretical foundation for developing efficient and sustainable green control technologies against soil-borne diseases.

## 1. Introduction

Soil-borne pathogens, including *Fusarium* spp., *Ralstonia solanacearum*, and *Phytophthora* spp., can persist in agricultural soils for a long time, causing devastating crop diseases, such as wilt disease and root rot, which incur substantial economic losses [1,2,3]. However, overreliance on chemical agents and pesticides introduces critical drawbacks: environmental contamination, enhanced pathogen resistance, and ecosystem disruption [4]. The rhizosphere, a soil microzone directly influenced by plant roots, harbors exceptionally diverse and dynamic microbial communities encompassing bacteria, fungi, archaea, protists, and viruses [5]. Moreover, it also harbors intricate networks of microbial communities and metabolites that are increasingly recognized as central determinants of plant health and resistance to soil-borne pathogens. Maintaining soil health represents a critical challenge for sustaining agricultural productivity under the pressure of modern intensive farming systems worldwide. Soil microorganisms serve as core regulators in preserving soil health, and one of their most representative functions is the suppression of soil-borne diseases [6]. Crucially, robust evidence confirms that microbiomes in a healthy rhizosphere effectively suppress soil-borne pathogens, forming naturally occurring ‘disease-suppressive soils’ [7,8]. Understanding the establishment, interaction networks, and ecological functions of plant microbial communities, as well as how plants regulate their microbial communities in response to external stresses, is fundamental for leveraging the plant microbiome to promote plant health and advance sustainable agriculture. The formation of plant microbial communities is influenced by a variety of biological and abiotic factors, with pathogen infections being a typical biological factor. Under pathogen invasion, plant roots recruit beneficial microbes by altering root exudates or releasing volatile organic compounds (VOCs), thereby facilitating the survival of their offspring or neighboring plants. This strategy is considered a plant’s ‘crying for help’. Beneficial microbes can serve as key components of the plant microbiome, promoting host health by inducing immune responses, secreting antibiotics, or competing with pathogens for resources and ecological niches. Thus, deciphering the assembly principles of rhizosphere microbiomes and their pathogen-inhibiting mechanisms and leveraging this knowledge for biocontrol strategies represents a pivotal pathway toward sustainable agriculture [9,10].

Several recent reviews have summarized the roles of rhizosphere microbiota in plant health, the formation of disease-suppressive soils, or the application of SynComs and prebiotics. However, an integrated framework that simultaneously considers plant genetic drivers (especially ‘*M* genes’), root exudate-mediated recruitment, multi-kingdom microbial interactions (including bacteria, fungi, protists, and phages), and translational strategies (SynComs, prebiotics, rotation, and organic amendments) remains lacking. Moreover, the potential to translate knowledge of microbiome-shaping genes into breeding programs, termed the ‘*M* gene → microbiome engineering → breeding’ axis, has not been explicitly articulated as a central concept in the previous literature. This review fills these gaps by (i) systematically presenting the ‘crying for help’ mechanism and its underlying signals; (ii) highlighting the emerging concept of ‘*M* genes’ that link plant genotype to rhizosphere microbiome assembly; (iii) integrating cross-domain interactions and network stability; and (iv) proposing a coherent pipeline from *M* gene identification to SynCom/prebiotics design and finally to resistant cultivar breeding. By doing so, we aim to provide a forward-looking framework that goes beyond descriptive accounts of microbial communities and offers actionable strategies for sustainable soil-borne disease management.

## 2. Rhizosphere Microbial Communities Assembly

**Figure 1:**
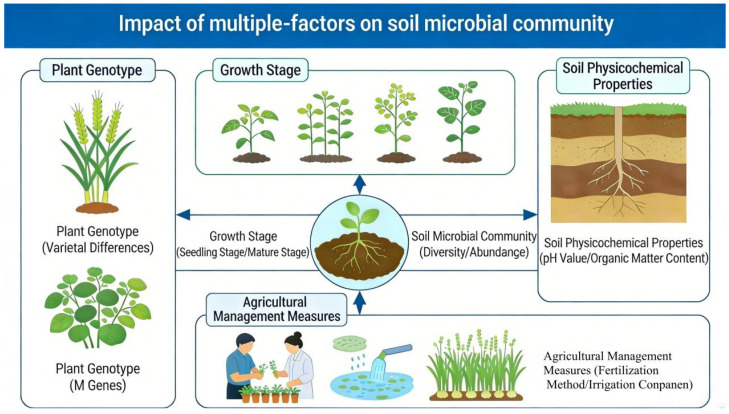
Multiple factors influencing rhizosphere microbial community.

The composition and function of the rhizosphere microbial community are not random, but are shaped by many factors such as plant genotype, development stage, soil physical and chemical properties and agronomic management measures (Figure 1) [11]. Among them, plant root exudates, as a key “bridge”, play a core role in driving microbial community assembly [12].

### 2.1. Assembly of Microbial Communities Through ‘Crying for Help’ Mediated by Root Exudates

When plants are infected or stressed by pathogens, they will actively change the composition of root exudates, release specific “cry for help” signals to the rhizosphere, and recruit beneficial microorganisms with antagonistic ability or those inducing plant resistance [13]. Many studies have revealed that plants can secrete specific primary and secondary metabolites, such as sugars, organic acids, amino acids, phenols, terpenoids, flavonoids, riboflavin, 3-hydroxyflavone, astaxanthin, and palmitic acid, to shape microbial communities in the rhizosphere, thereby enhancing their plant disease resistance (Table 1).

For example, after tomato was infected by *R. solanacearum*, malic acid secreted by roots recruited *Sphingomonas* to reduce bacterial wilt disease [14]. After cucumber was infected by *Fusarium oxysporum*, the secretion of four organic acids (citric acid, pyruvate acid, succinic acid, and fumarate) increased, recruiting beneficial microorganisms *Comamonadaceae* and *Xanthomonas* to produce anti-pathogenic substances and increase disease resistance [15]. Ma et al. [16] observed that methyl ferulate was secreted in greater quantities in the tobacco, recruiting disease-suppressive rhizosphere microbes, such as *Bacillus* (the relative abundance of these microbes increased from 4.69% to 13.79%), thereby increasing black shank disease resistance. Another study demonstrated that salicylic acid could specifically increase some beneficial rhizosphere species that can confer resistance against watermelon *Fusarium* wilt, such as *Rhodanobacter*, *Sphingomonas*, and *Micromonospora* [17].

In addition to the root exudates secreted by plants themselves, the root exudates secreted by neighboring or other plants can also reshape the microbial communities, thereby resulting in enhanced plant disease suppression. For example, in the cabbage–buckwheat rotation system, buckwheat root-secreted flavonoids 6,7,4′-trihydroxyisoflavone and 7,3′,4′-trihydroxyflavone, as key drivers of microbial community restructuring, synergistically enhanced the efficacy of a synthetic microbial community, boosting clubroot disease suppression by 34% in greenhouse trials [18]. In an intercropping system, the root exudate taxifolin secreted by potato onion (*Allium cepa* var. *aggregatum*) recruits beneficial bacteria such as *Bacillus* sp. to protect tomato plants against *Verticillium dahliae* [19]. Similarly, Zhu et al. [20] demonstrated that ginger root exudates, including sinapyl alcohol and 6-gingerol, greatly promoted the proliferation and colonization of *Burkholderia* sp. in the chrysanthemum rhizosphere, conferring enhanced *Fusarium* wilt disease suppression. In addition, the coexistence of five phenolic acids in the maize rhizosphere showed strong synergistic antimicrobial activity against the infection of *Phytophthora sojae* on soybean [21]. Although an increasing number of root exudates are involved in recruiting disease resistance-associated microbes to enhance plant disease resistance, little is known about the ternary link of plant genes, root exudates, and disease resistance-associated microbes [12].

### 2.2. Assembly of Microbial Communities Influenced by Host Genotype and ‘M Genes’

Host genotype plays a pivotal role in shaping the rhizosphere microbiome, which directly impacts the plant’s susceptibility or resistance to soil-borne diseases. Resistant cultivars often assemble distinct microbial communities enriched with beneficial taxa such as *Pseudomonas*, *Bacillus*, and *Sphingomonas*, which contribute to disease suppression through mechanisms like antibiosis, competition, and induced systemic resistance. In contrast, susceptible cultivars tend to harbor more pathogenic microbes and less diverse beneficial communities. This differentiation is largely driven by plant genetic factors, including “*M* genes” (microbiome-shaping genes), which regulate root exudate profiles, such as the secretion of flavonoids, organic acids, and nucleotides that selectively recruit protective microbes. For instance, when resistant tomato varieties were infected by *Fusarium oxysporum*, the fusaric acid promoted the recruitment of beneficial microbial taxa led by *Sphingomonas* sp. Sm12 and prevented *Fusarium* wilt by antagonizing and inducing host resistance [22]. Maize cultivars resistant to stalk rot reshaped the microbiota and recruited *Bacillus* species with three phenotypes against *Fusarium graminearum*, including niche pre-emption, potential secretion of antimicrobial compounds, and no inhibition to alleviate pathogen stress [23]. Among them, *Bacillus* isolates that do not directly suppress pathogen infection inhibited pathogen growth by inducing the synthesis of berberine, an isoquinoline alkaloid. Yue et al. [24] found that a wild wheat cultivar was able to harness rhizosphere microorganisms carrying nitrogen transformation (i.e., nitrification and denitrification) and phosphorus mineralization pathways, whereas rhizobiomes carrying inorganic nitrogen fixation, organic nitrogen ammonification, and inorganic phosphorus solubilization genes were recruited by the release of root exudates from domesticated wheat.

**Figure 2:**
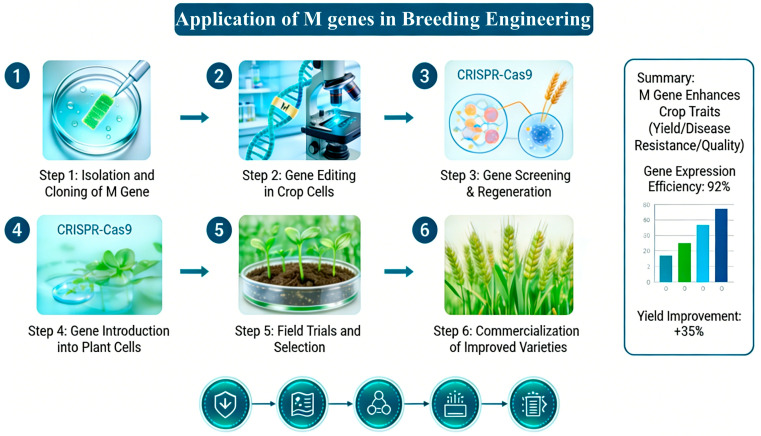
The pipeline for harnessing *M* genes as a breeding target.

The capacity to shape the microbiome through specific genes has previously been demonstrated in various plants, including *Arabidopsis*, barley, tomato, cabbage, cucumber, maize and rice [25]. These microbiome-shaping genes have been reported to contribute to promoting host plant performance and resilience against diverse stresses, including balancing the growth–defense trade-off, coping with nitrogen deficiency, defending against soilborne pathogens and enhancing nitrogen acquisition. A common characteristic of these genes is the dependence of their shaping effect on the root or rhizosphere microbiota. Therefore, these genes that can shape the microbiome are called ‘microbiome-shape genes’ or ‘*M* genes’ for short. Yang et al. [26] found that RIN, the key transcription factor of tomato fruit ripening, recruited actinomycetes to inhibit tomato bacterial wilt by promoting the secretion of riboflavin and 3-hydroxyflavone. The *Fusarium* wilt resistance gene FOC1 was transferred into the susceptible variety to breed resistant varieties with a highly similar genetic background. Compared with the susceptible variety, *Pseudomonas brasciarum* NA13, a dominant bacterium recruited by resistant varieties, can significantly improve the resistance of cabbage varieties susceptible to *Fusarium* wilt by directly inhibiting the growth of *Fusarium oxysporum* f. sp. *conglutinans* (FOC) and triggering the root defense mechanism [27]. Therefore, exploring the possibility of different functional genes in plants becoming ‘*M* genes’ and carrying out the corresponding microbial breeding project will open up a new way for more effective utilization of microbial communities for disease prevention and control (Figure 2).

### 2.3. Assembly of Microbial Communities Driven by Soil Physicochemical Properties

Soil physicochemical properties form a critical environmental filter that deterministically shapes the assembly, diversity, and function of the rhizosphere microbial community. Recent studies continue to highlight soil pH as a master regulator, exerting a dominant influence on bacterial community composition and niche differentiation by altering nutrient solubility and microbial cellular physiology. For instance, in flue-cured tobacco, soil acidification was linked to reduced microbial diversity and altered community structure, often favoring pathogenic fungi while suppressing beneficial bacteria [28]. Similarly, in pepper soils, pH showed strong correlations with the abundance of specific bacterial phyla like *Actinobacteria* and *Planctomycetes* [29]. In soybean, pH was identified as the strongest predictor of microbial community structure, showing the highest correlation with beta diversity [30]. In addition, the availability of macronutrients like nitrogen (N), phosphorus (P), and potassium (K) is equally crucial. Available phosphorus (AP) and potassium (AK) were significantly higher in intercropped pepper–maize systems, which corresponded to increased microbial diversity and enrichment of beneficial taxa like *Sphingomonadales* [29]. In soybean, potassium was the most important macronutrient distinguishing high-yield from low-yield states, showing a strong positive correlation with yield and microbial modules beneficial for plant growth [30]. In *Ligusticum chuanxiong*, the severity of root rot was significantly influenced by soil available nitrogen (AN), AP, and AK, which, in turn, shaped the rhizosphere microbiome, affecting the abundance of biocontrol agents like *Trichoderma* and *Bacillus* [31]. Soil organic matter (SOM) serves as a critical carbon and energy source for microbes. Higher SOM content generally promotes microbial abundance and diversity. In *Cerasus humilis* orchards, SOM content increased with planting years and was positively correlated with the abundance of beneficial bacteria like *Acidobacteria* and *Proteobacteria* [32]. Conversely, continuous tobacco cropping led to a decline in SOM, which was associated with a reduction in microbial diversity and a shift towards a more pathogenic community [33]. Furthermore, the interplay of these physicochemical properties creates distinct ecological niches that select for specific microbial taxa. For example, soil cation exchange capacity (CEC) and metal content can also exert selective pressures, as demonstrated in cherimoya and lucuma soils, where metal concentrations correlated with shifts in microbial diversity [34]. In summary, soil physicochemical properties act as environmental filters that govern the assembly of rhizosphere microbial communities by influencing nutrient availability, creating chemical gradients, and modulating microbial interactions. Maintaining balanced soil properties is, therefore, crucial for promoting beneficial microbiomes and ensuring plant health.

### 2.4. Assembly of Microbial Communities Affected by Agricultural Management

Enhancing the disease-suppressive capacity of the plant rhizosphere is an ideal approach for reducing pesticide use in sustainable agriculture. Consequently, employing specific agricultural management practices to manipulate the soil microbiome for the creation of disease-suppressive soils represents a challenging yet promising area of exploration. Studies have shown that soil fumigation and reductive soil disinfestation (RSD) are relatively effective methods for directly modulating the soil microbial community to suppress soil-borne diseases. The combined application of fumigation, RSD, and various amendments such as microbial organic fertilizers and microbe-enriched biochar-based fertilizers can significantly enhance the suppression of soil-borne diseases. These strategies have been widely used to control *Fusarium* wilt in crops like watermelon, eustoma, pepper, banana, and *Salvia miltiorrhiza* [35,36,37]. Additionally, practices like crop rotation, intercropping, and the incorporation of plant residues are common and powerful measures for manipulating the soil microbiome to control soil-borne diseases. Compared with crop rotation, monoculture systems often lead to a competitive disadvantage among soil microbes for limited root exudate resources, resulting in the loss of key rhizosphere microorganisms. However, supplementing these missing microbial consortia can restore the rhizosphere’s ability to suppress root rot in monoculture systems [38]. For example, when pepper and eggplant were rotated with banana, distinct core microbiomes were formed under different crop rotations [39]. In the case of intercropping potato onion with tomato, the root exudates of potato onion, particularly taxifolin (a flavonoid), alter the chemical properties of tomato root exudates. This directly inhibits the growth of *Verticillium dahliae* and indirectly promotes the colonization of *Bacillus* spp. on tomato roots, inducing systemic resistance to control *Verticillium* wilt [19]. When aboveground and belowground residues of pineapple were added to soil with a high incidence of banana *Fusarium* wilt, they significantly enriched *Aspergillus fumigatus* and *Fusarium solani*. These fungi reduce the abundance of pathogens and suppress disease through direct antibiotic production and indirect nutrient competition [40]. Thus, regulating the establishment of disease-suppressive microbiomes through agricultural practices offers diverse options for precisely manipulating the plant microbiome.

## 3. Core Beneficial Microbes and Their Disease Suppression Mechanisms

**Figure 3:**
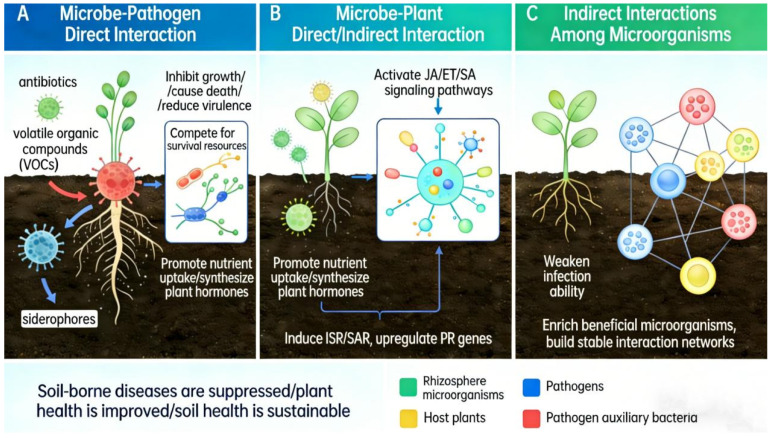
The main pathways and mechanisms of rhizosphere microbes controlling soil-borne diseases in crops. VOCs: volatile organic compounds; SAR: systemic acquired resistance; ISR: induced systemic resistance; JA: jasmonic acid; SA: salicylic acid; ET: ethylene.

Currently, a growing body of research confirms that plant root-associated microorganisms, a general term for rhizosphere and root endophytic microorganisms, play crucial roles in plant–pathogen interactions through various mechanisms. These include the production of antibiotics [40], competition for ecological niches and nutrients [40], suppression of pathogen helpers [41], induction of host disease resistance [42], and promotion of host antibiotic production [23]. It is noteworthy that the root-associated microbial communities recruited by plants often function as a whole through multifunctional synergistic interactions and exhibit a certain degree of functional redundancy. This microbiota forms a functional alliance through complex interspecific interactions to jointly resist pathogens. Among them, some core microbes have been repeatedly proven to play a key role in disease suppression. These microbes employ a multifaceted approach to suppress soil-borne diseases, primarily through the production of antimicrobial compounds such as phenazines, siderophores, and volatile organic compounds (VOCs) that directly inhibit pathogen growth by targeting essential cellular processes like DNA topoisomerase IV, leading to DNA damage and cell death. They also modulate the rhizosphere microbiome by enriching beneficial microbes, such as *Bacillus* and *Trichoderma*, and suppress pathogens through competitive exclusion and iron competition. Additionally, they induce systemic resistance in plants by upregulating defense-related genes involved in jasmonic acid (JA)/ethylene (ET) and salicylic acid (SA) signaling pathways, enhancing plant innate immunity [43]. This combined action results in reduced disease incidence, improved plant growth, and sustained soil health. The main pathways and mechanisms of rhizosphere microbes controlling soil-borne diseases in crops are presented in Figure 3.

### 3.1. Bacteria

*Pseudomonas* spp.: In tomato plants, *Pseudomonas* consortia significantly suppressed bacterial wilt caused by *R. solanacearum* primarily through siderophore-mediated iron competition under iron-limited conditions, where siderophores accounted for 73.81% of pathogen growth inhibition in vitro, and stronger siderophore-driven interactions within consortia directly reduced pathogen abundance and disease severity in greenhouse trials [44]. Moreover, *Pseudomonas rhizophila* CTR8 isolated from healthy cotton roots significantly suppressed *Verticillium* wilt caused by the fungus *Verticillium dahliae*. Its primary mechanisms include the production of specific VOCs, namely, dimethyl trisulfur compounds (causing 100% mycelial growth inhibition), 2-undecanone (56.33% inhibition), and 1-decanol (13.67% inhibition), which directly inhibited the pathogen and downregulated its virulence genes, alongside activating the plants’ JA/ET-dependent induced systemic resistance [45]. Additionally, the combined application of *Trichoderma asperellum* + *Pseudomonas fluorescens* + arbuscular mycorrhizal fungi (AMF) controlled the root rot disease caused by *Rhizoctonia solani* through direct antagonism via metabolite production and enrichment in the rhizosphere microbiome, achieving reduced disease severity and improved plant vigor in field experiments [46]. Furthermore, *Pseudomonas chlororaphis* controlled various soil-borne diseases caused by *Fusarium* in multiple plants through production of phenazines like phenazine-1-carboxamide (PCN), which directly bind to bacterial topoisomerase IV, inhibiting decatenation activity and causing DNA damage and cell death in pathogens, achieving high inhibition rates, such as 100% mycelial growth inhibition, and reduced disease incidence in laboratory and field settings [47]. *Pseudomonas fluorescens* LJPf01 controlled bacterial wilt caused by *R. solanacearum* in tomato through induction of plant defense pathways, such as upregulation of PR genes and phenylpropanoid biosynthesis, modulation of rhizosphere microbial communities, and production of inhibitory metabolites, achieving a biocontrol efficiency of up to 76.08%, significantly increased plant height, root length and stem thickness from 96.47 cm, 13.14 cm and 9.06 mm to 105.49 cm, 15.48 cm and 11.75 mm, respectively, and improved chlorophyll content [48].

*Bacillus* spp.: *Bacillus velezensis* RC116 effectively controlled tomato bacterial wilt caused by *R. solanacearum* and *Fusarium* wilt caused by *Fusarium oxysporum* by secreting the extracellular enzymes protease and amylase, siderophores, and indoleacetic acid (IAA), while possessing biocontrol genes for antimicrobial compound synthesis [49]. *B. velezensis* ZF481, encapsulated in chitosan or carrageenan macrospheres for slow release, targeted clubroot disease caused by *Plasmodiophora brassicae* in Chinese cabbage and pak choi by producing antifungal metabolites and enhancing soil nutrient availability, such as phosphorus and potassium [50]. Similarly, fengycin, produced by *B. subtilis* XF-1, enhanced the activation of plant defense via JA and SA signaling pathways, thus controlling the Chinese cabbage clubroot [51]. Moreover, *Bacillus* species in resistant maize cultivars combated corn stalk rot caused by *Fusarium graminearum* through inducing host alkaloid biosynthesis and other metabolic defenses, such as berberine expressed by TYDC1, which inhibited pathogen growth. This reshaped the rhizosphere microbiome, reducing disease incidence in resistant cultivars [23].

*Streptomyces* spp.: Alper et al. [52] isolated *Streptomyces* from the rhizosphere soil of olive trees, whose inhibitory effect on *Aspergillus niger* could achieve the same effect as ketoconazole. Sujarit et al. [53] found that three kinds of *Streptomyces* (*Streptomyces noursei* CMU-AB21, *Streptomyces sioyaensis* CMU-AB83 and *Streptomyces palmae* CMU-AB204^T^) all had strong antifungal activity. Among them, *S. palmae* CMU-AB204^T^ can reduce the incidence of stem base rot of palm by 75.8–81.6%. Nguyen et al. [54] showed that the growth of *Fusarium verticillioides* could be reduced to 60% by adding *Streptomyces* isolated from soil, and the toxins Fumonisin B1 and Fumonisin B2 secreted by eumycetes could be reduced by 87.5–98.2% after 6 days of cultivation. *Streptomyces* sp. JCK-6116 produced rimocidin compounds that disrupt fungal cell membranes by binding to ergosterol, effectively suppressing *Fusarium* wilt and damping-off caused by *Fusarium oxysporum* and *Rhizoctonia solani* in cucumbers [55]. *Streptomyces* R02, a strain from the tomato rhizosphere, enhanced biocontrol efficacy of bacterial wilt caused by *R. solanacearum* through synergistic interactions with native rhizosphere microbes, such as *Stenotrophomonas maltophilia* CSC98 and *Paenibacillus cellulositrophicus* CSC13 [56]. This interaction induced the production of erythromycin E, a macrolide antibiotic that directly inhibited the pathogen by disrupting bacterial protein synthesis. A recent study demonstrated that *Streptomyces* specifically primed pepper root biosynthesis of 6-nitrocoumarin, a nitrated coumarin derivative exhibiting potent broad-spectrum antioomycete activity [57]. Crucially, this *Streptomyces*-recruited metabolite functions as a keystone rhizosphere signal, selectively enriching beneficial bacterial consortia while directly disrupting the ABC transporters of pathogens. The restructured microbiome synergistically amplifies plant systemic resistance through upregulating defense genes and enhancing root immunity. Furthermore, *Streptomyces* was demonstrated to be mainly negatively correlated with the abundance of the pathogen *Fusarium pseudograminearum* causing wheat crown rot [58].

*Burkholderia* spp.: In recent research, intercropping chrysanthemum with ginger suppressed *Fusarium* wilt disease caused by *Fusarium oxysporum* f. sp. *chrysanthemi* (Foc) by modulating the rhizosphere microbiome through root exudate-mediated interactions. The suppression mechanism is achieved as specific compounds in ginger root exudates (e.g., sinapyl alcohol and 6-gingerol) stimulate chrysanthemum roots to recruit and enrich beneficial *Burkholderia* species, which, in turn, inhibit the pathogen via antagonism and induce systemic resistance in the host plant [20]. Shen et al. [59] identified antagonistic bacteria for biocontrol against cucumber *Fusarium* wilt caused by *Fusarium oxysporum* f. sp. *cucumerinum*. Among eight strains, *Burkholderia cepacia* L1-3 and *Sphingomonas paucimobilis* L5-4 showed the strongest disease suppression, with 42.67% and 36.67% efficacy and growth-promoting traits. The mechanism includes direct antagonism (inhibiting pathogen growth), enhancing plant immunity (elevating antioxidant enzymes like POD and SOD), and improving nutrient uptake (solubilizing phosphorus, producing IAA, and fixing nitrogen). Applied as microbial agents, these strains increase cucumber seedling vigor, chlorophyll content, and root activity, demonstrating their potential as eco-friendly alternatives to chemical fungicides. Moreover, another study demonstrated that rewilding the rhizosphere microbiome of cultivated *Coptis chinensis* by introducing key ancestral bacteria, particularly strains of *Paraburkholderia* (*P. nemoris* and *P. phytofirmans*), played an essential role in suppressing root rot disease caused by fungal pathogens like *Ilyonectria robusta* and *Fusarium solani*. The control mechanism involves the direct antagonistic activity of the *Paraburkholderia* isolates, which inhibit pathogen growth and spore germination, coupled with their plant growth-promoting properties, such as nitrogen fixation and phosphate solubilization, which enhance overall plant health and immune responses [60].

*Sphingomonas* spp.: *Sphingomonas* confers effective suppression against diverse soil-borne diseases via multiple consistent mechanisms. In wheat, increased *Sphingomonas*, such as *Sphingomonas azotifigens*, reduced infection by Wheat yellow mosaic virus (WYMV) transmitted by *Polymyxa graminis* by upregulating auxin and cytokinin signaling pathways for growth promotion and modulating jasmonic acid (JA) and salicylic acid (SA) pathways to activate host systemic resistance, with a significant reduction in viral load [61]. For potato, *Sphingomonas* enrichment in the rhizosphere, induced by *Pseudomonas frederiksbergensis* FC-17-fortified bio-organic fertilizer, mitigates bacterial wilt triggered by *R. solanacearum* through rhizosphere microbiome restructuring, ecological niche competition, and negative correlation with disease index [62]. In Chinese cabbage, high *Sphingomonas* abundance serves as a key biomarker to resist clubroot caused by *Plasmodiophora brassicae*, with higher relative abundance in healthy soils than diseased soils and potential involvement in suppressing pathogen colonization [63]. In tomato, augmented *Sphingomonas* alleviates *Fusarium* wilt caused by *Fusarium oxysporum* f. sp. *Lycopersici* and bacterial wilt caused by *R. solanacearum* through multiple mechanisms: direct antagonism against pathogens, induced systemic resistance (ISR) via upregulating defense-related genes, synergistic interactions with other beneficial microbes, and recruitment by root exudates such as succinic acid [14].

In conclusion, it can be seen that multiple bacterial genera suppress soil-borne diseases via overlapping and synergistic mechanisms. *Pseudomonas* spp. employ siderophore-mediated iron competition, volatile organic compounds (e.g., dimethyl trisulfur), and JA/ET-dependent induced resistance. *Bacillus* spp. secrete antimicrobial metabolites (e.g., fengycin), activate JA/SA signaling, enhance nutrient availability, and trigger host alkaloid biosynthesis (e.g., berberine). *Streptomyces* spp. produce antifungal compounds (rimocidin and 6-nitrocoumarin), synergize with native rhizobacteria, and prime plant roots for defense. *Burkholderia* spp. directly inhibit fungal pathogens, induce systemic resistance via root exudates, promote plant growth (P solubilization and N fixation), and boost antioxidant enzymes. *Sphingomonas* spp. antagonize pathogens, activate JA/SA pathways, restructure the rhizosphere microbiome, and serve as biomarkers of soil health. Collectively, these core bacteria achieve disease suppression through direct antagonism, resource competition, immune activation, and microbiome modulation.

### 3.2. Fungi

*Trichoderma* spp.: *Trichoderma asperellum* FJ035 controls cucumber *Fusarium* wilt caused by *Fusarium oxysporum* f. sp. *cucumerinum* by restructuring the rhizosphere microbiome, promoting the colonization of beneficial bacterial consortia, such as *Streptomyces* and *Pseudomonas*, and inducing ROS bursts and systemic resistance via upregulating antioxidant enzyme and defense-related gene expression [64]. *Trichoderma harzianum* T891 suppresses red kidney bean root rot incited by *Fusarium oxysporum* by enhancing plant antioxidant enzyme (SOD, POD, and CAT) activity, reducing pathogen abundance, and enriching beneficial rhizobacteria to stabilize the microbial co-occurrence network [65]. *Trichoderma virens*, a component of synthetic consortia derived from wheat rhizosphere, inhibits wheat crown rot caused by *Fusarium pseudograminearum* by direct antagonism against the pathogen and synergizing with other beneficial microbes to activate salicylic acid, jasmonic acid, and abscisic acid-mediated defense pathways [58]. Additionally, *Trichoderma*-based bioorganic fertilizers mitigate soil-borne diseases by modulating native microbial communities, competing for ecological niches with pathogens, and inducing plant systemic resistance, thereby forming a protective rhizosphere microbiota against diverse soil-borne pathogens [66].

Arbuscular mycorrhizal fungi (AMF): AMF serve as pivotal regulators of rhizosphere microbial dynamics, enabling plants to combat soil-borne diseases through multifaceted and synergistic mechanisms. AMF establish extensive extraradical mycelial networks that modify soil microhabitats, selectively recruiting beneficial microbes such as *Pseudoxanthomonas*, *Sphingomonas*, and *Bacillus* to enhance antagonistic activities against pathogens via antibiotic secretion and niche competition [67]. They modulate root exudate profiles and trigger mycorrhiza-induced resistance (MIR), activating salicylic acid (SA)- and jasmonic acid (JA)-mediated defense pathways and upregulating pathogenesis-related (PR) genes to reinforce plant immunity [68]. Nursery-stage AMF inoculation induces long-lasting shifts in rhizobacterial metabolic functions, including enhanced carbohydrate degradation, stress-responsive metabolite biosynthesis, and reduced energy-intensive processes, which persist throughout the crop cycle [69]. AMF also promotes common mycorrhizal networks (CMNs) that facilitate interplant defense signal transmission and nutrient sharing, while increasing the complexity and stability of rhizosphere microbial networks. In intercropping systems, AMF further amplify disease suppression by boosting microbial diversity, optimizing nutrient uptake, and enhancing the expression of resistance-related genes [68]. These advances highlight AMF as key drivers of rhizosphere microbiome engineering, offering sustainable and eco-friendly alternatives to chemical fungicides in agricultural disease management.

*Chaetomium* spp: *Chaetomium* species exhibit robust biocontrol potential against diverse soil-borne diseases across multiple crops, with distinct strains targeting specific pathogens through synergistic mechanisms. Specifically, *Chaetomium cupreum* CT07 combats *Rhizoctonia solani*-induced damping-off in cruciferous vegetables, such as *Brassica juncea* and *Brassica rapa* var. *Integrifolia*, via antifungal metabolite production and nutrient competition, achieving 83.4% inhibition in dual cultures, while concurrently promoting plant growth, indicating iron competition can dominate over other specific mechanisms in this system [70]. Meanwhile, *Chaetomium globosum* 12XP1-2-3 controls wheat *Fusarium* crown rot caused by *Fusarium pseudograminearum* through mycelial antagonism, fermentation broth-mediated suppression of spore germination and mycelial growth, induced systemic resistance, and favorable modulation of the rhizosphere microbiome [71]. For eggplant, *Chaetomium globosum* CEF-082 inhibits three key soil-borne pathogens, *Verticillium dahliae*, *Fusarium solani* and *Phomopsis vexans*, with its 10% fermentation broth suppressing spore germination by 67.7–70.6% and delivering 39.4–95.2% control efficacy in pot trials [72]; notably, this same strain also alleviates cotton *Verticillium* wilt and mitigates continuous cropping obstacles by regulating soil microbial communities, enhancing soil enzyme activities, and improving nutrient balance [73]. Additionally, *Chaetomium globosum* NP2 mitigates tobacco root rot by modifying rhizosphere microbiome structure and inhibiting pathogen colonization [74].

In summary, fungal biocontrol agents, including *Trichoderma* spp., arbuscular mycorrhizal fungi (AMF), and *Chaetomium* spp., suppress diseases through diverse mechanisms. *Trichoderma* spp. directly antagonize pathogens, restructure the rhizosphere microbiome to enrich beneficial bacteria, induce systemic resistance and oxidative bursts, and synergize with bio-organic fertilizers to activate SA/JA/abscisic acid pathways. AMF form extraradical mycelial networks that recruit beneficial bacteria, trigger mycorrhiza-induced resistance (MIR) via SA/JA signaling and PR gene upregulation, induce long-lasting metabolic shifts in rhizobacteria, and establish common mycorrhizal networks (CMNs) for interplant defense signaling and nutrient sharing. *Chaetomium* spp. produce antifungal metabolites (e.g., 83.4% inhibition against *Rhizoctonia solani*), induce systemic resistance, modulate rhizosphere microbiome structure and enzyme activities, and exhibit broad-spectrum activity against *Verticillium*, *Fusarium*, and *Phomopsis*. Together, these fungi employ direct antagonism, microbiome engineering, induced plant resistance, and network-mediated cooperation to enhance rhizosphere disease suppression.

## 4. Microbial Interaction Network and Community Stability

Rhizosphere disease suppression depends not only on a single or a few beneficial microorganisms, but also on the composition, diversity and stability of the whole microbial community. The interactions between microorganisms, such as symbiosis, antagonism and predation, form a complex network structure.

### 4.1. Network Structure and Disease Inhibition

A healthy plant rhizosphere usually has a more complex and highly connected microbial co-occurrence network, which contains more negative correlation connections, which may represent antagonism or competition. The key species, namely, keystone taxa or hub taxa in the network, are very important to maintain the stability of community structure and function. For example, the rhizosphere microbial network of disease-resistant wheat is more complex and robust than that of susceptible varieties [24]. The microbial network in disease-resistant soil has a higher density and a more negative correlation [1,75].

### 4.2. Functional Redundancy and Resilience

The functional redundancy of microbial communities, namely, different species performing similar functions, can enhance the resilience of communities against environmental disturbances, such as drought and acidification, and pathogen invasion. Soil acidification will destroy the bacterial community structure and reduce its disease-inhibiting ability, partly because of the decrease in the abundance of key functional groups, such as microorganisms involved in sulfur metabolism, and the damage to the stability of the interaction network [76].

### 4.3. The Role of Protozoa and Viruses

Predatory protozoa can regulate the structure and function of bacterial communities through predation and indirectly affect the abundance of pathogens. For example, the abundance of bacteria-eating protozoa, such as *Cercomonas*, is negatively correlated with the density of *R. solanacearum*, and its community structure can predict the health status of plants [77]. Phage deeply influences the dynamics and functions of bacterial communities by lysing host bacteria, including pathogens, and mediating horizontal gene transfer [78].

**Figure 4:**
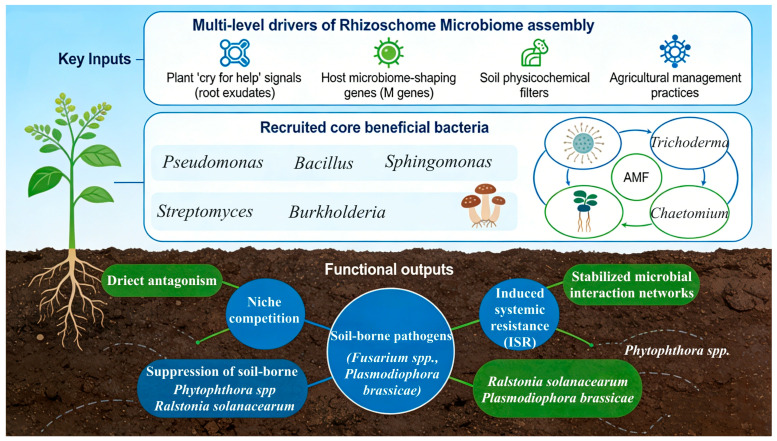
Multi-level drivers of rhizosphere microbiome assembly and disease suppression.

Collectively, the assembly of rhizosphere microbial communities is shaped by root exudates, host genotype, soil properties, and agricultural practices, while core beneficial microbes (e.g., *Pseudomonas*, *Bacillus*, *Streptomyces*, *Trichoderma*, and AMF) suppress soil-borne pathogens through antibiosis, competition, induced systemic resistance, and modulation of microbial interaction networks (Figure 4).

## 5. Control Strategy and Application Based on Rhizosphere Microbiota

Based on the understanding of the disease suppression mechanism of rhizosphere microbiota, a variety of strategies for preventing and controlling soil-borne diseases by using microbiota are being developed and applied.

### 5.1. Synthetic Microbial Community (SynCom)

SynCom refers to the combination and application of microorganisms (usually including bacteria and fungi) with specific functions to simulate or optimize the functions of natural communities. SynCom usually has a more stable and efficient inhibitory effect than a single strain. For example, SynCom, composed of 16 core bacteria, can effectively inhibit watermelon *Fusarium* wilt, and its effect is better than that of single bacteria, and it can reshape rhizosphere microbial groups and enrich beneficial *Pseudomonas* [79]. SynCom, which is composed of *Bacillus* and *Streptomyces*, significantly enhances the control effect of tomato bacterial wilt through the synergy among its members, such as metabolite-induced antibiotic synthesis [56]. SynCom, composed of five strains of *Bacillus* and five strains of *Pseudomonas*, inhibited strawberry wilt by improving soil versatility, nutrient cycle and enzyme activity, and enriching beneficial microorganisms such as *Burkholderia*, and showed a lasting legacy effect [80].

### 5.2. Rhizosphere Prebiotics

Rhizosphere prebiotics are screened compounds (usually plant or microbial metabolites) that can specifically stimulate the growth or activity of beneficial microorganisms but not promote the growth of pathogens. For example, metabolites such as ribose, lactic acid, xylose, mannose, maltose, gluconolactone and ribitol identified from the rhizosphere of healthy tomatoes can selectively promote the growth of beneficial bacteria such as *Bacillus* and *Lysobacter* and inhibit bacterial wilt, and they can effectively control tomato bacterial wilt when applied to soil [81]. Specific nucleotides enriched in the citrus rhizosphere, such as adenosine, can recruit beneficial microorganisms and enhance plant heat resistance and disease resistance [82].

### 5.3. Legacy Effect of Soil Microorganisms and Rotation/Intercropping

The previous crop creates a specific rhizosphere microbial group through root exudates and residues, which has a legacy effect on the health of the next crop. Reasonable rotation or intercropping can take advantage of this effect to prevent and control soil-borne diseases. For example, buckwheat–cabbage rotation significantly reduces the incidence of cabbage clubroot, and its mechanism is that flavonoids secreted by buckwheat roots enrich beneficial microorganisms, including *Microbacterium*, *Stenotrophomonas* and *R. solanacearum*, which can inhibit pathogens [18]. Ginger–chrysanthemum intercropping stimulates the root system of chrysanthemum to release specific metabolites through ginger root exudates, such as sinapine and 6-gingerol, which promote the colonization of *Burkholderia* in the root system of chrysanthemum and the formation of biofilm, thus inhibiting chrysanthemum wilt [20].

### 5.4. Organic Improvement and Soil Health Management

The application of organic fertilizer and compost can improve soil physical and chemical properties, increase microbial diversity, enrich beneficial microorganisms and enhance soil disease inhibition. For example, the application of bio-organic fertilizer can significantly reduce the incidence of tomato bacterial wilt, and its effect is related to the change in rhizosphere microbial community structure by the enrichment of *Pseudomonas* and the enhancement of function, for example, the increase in antibiotic synthesis gene abundance [8,83]. Furthermore, long-term application of organic fertilizer is helpful to maintain a healthy rhizosphere microbial network by regulating bacterial composition and soil metabolic processes [2,84].

### 5.5. Host Plant Breeding and Engineering

Enhancing plant disease resistance in both the plant pathway and the rhizobiome pathway is undoubtedly a shared goal for plant breeders and microbiologists. For microbiologists, recognizing the contribution of the rhizobiome pathway to plant disease resistance is a prerequisite for the precise and efficient utilization of rhizosphere microbes in promoting plant disease resistance [12]. In the context of disease resistance breeding, the microbiome-shaping genes enable both the enrichment of disease-suppressive microbiota and a direct disease-suppressive effect. Current breeding strategies based on the accumulation of resistance genes are increasingly challenged by rapidly evolving pathogens [25]. By contrast, harnessing microbiome-shaping genes may facilitate the breeding of crop varieties that are more broad-spectrum and durable in disease resistance. Therefore, crop varieties that can effectively recruit and maintain beneficial rhizosphere microorganisms should be bred or transformed, for example, using GWAS and other technologies to identify plant gene loci related to root exudate composition and microbial recruitment ability and guide disease-resistant breeding [12].

## 6. Challenges and Future Directions

Although rhizosphere microbiotas show great potential in controlling soil-borne diseases, it still faces many challenges. First of all, field stability and predictability are often lacking. Synthetic microbial communities (SynComs) or prebiotic strategies that perform effectively in laboratories or greenhouses frequently become unstable in complex field environments. Environmental factors (e.g., soil type, climate, and agricultural practices), competition from indigenous microbial communities, and dynamic shifts in pathogenic bacterial communities collectively influence the colonization efficiency and functional expression of introduced microorganisms or metabolites. Secondly, the depth of mechanistic understanding is insufficient. The molecular mechanisms underlying complex signal communication among plants, beneficial microbes, and pathogens, such as root exudate–microbial receptor recognition and microbial secondary metabolite-mediated plant immune activation, remain to be deeply dissected. Moreover, the functional redundancy and synergistic mechanisms at the microbiome level need to be clearly clarified. Thirdly, the complexity of cross-domain interactions is understudied. The comprehensive contributions of interactions across microbial domains (e.g., bacteria–fungi cross-talk, protist predation, and phage-mediated regulation) to disease suppression require systematic investigation. In addition, standardization and large-scale application are urgently needed. The production, storage, transportation, application techniques, and efficacy evaluation systems of SynComs require standardization, and the screening, dosage, and application modes of prebiotics also need optimization, with cost-effectiveness being the key constraint for large-scale field deployment. Finally, studies on rhizosphere viruses, especially the structure, function, and disease-suppressive roles of bacteriophages in the rhizosphere, represent an emerging and promising research direction [78].

To further advance the development and application of rhizosphere microbiome-based disease control strategies, future research should focus on the following key directions. Firstly, in-depth analysis of the molecular mechanisms underlying rhizosphere ‘crying for help’ signals is urgently needed, including the identification of key signal molecules and their corresponding microbial receptors, as well as clarification of the signaling pathways that regulate the synthesis and secretion of these signals after plants perceive pathogen invasion. Next, the design of smarter and more stable synthetic microbial communities (SynComs) should be achieved based on a systematic understanding of microbial interaction networks and functional redundancy. Such SynComs should be capable of self-assembly, environmental adaptation, and sustained functional expression, and ideally include cross-domain members such as bacteria, fungi, and bacteriophages. Subsequently, development of precision delivery systems is required to protect introduced microorganisms or bioactive metabolites using nanomaterials and biomaterials, thereby enabling targeted delivery and controlled release in the rhizosphere. Then, integration with host breeding should be strengthened by using genome editing and other biotechnologies to create new crop varieties that can more efficiently recruit and maintain beneficial microbial communities, for example, by optimizing root system architecture and root exudate profiles. Eventually, integration of multiple strategies is essential to combine synthetic microbial community inoculation with soil health management (e.g., organic amendment, crop rotation, and intercropping), disease-resistant variety breeding, and physical control measures, thereby forming a comprehensive and efficient disease prevention and control system. If possible, field validation and modeling prediction should be enhanced through large-scale field trials across diverse ecological regions and crop systems to evaluate long-term efficacy and ecological risks. Meanwhile, predictive models for disease occurrence and control efficacy should be developed using big data and artificial intelligence to support precise and intelligent management of soil-borne diseases.

## 7. Conclusions

The rhizosphere microbiota serves as a natural barrier for plants against soil-borne diseases. The ‘crying for help’ mechanism, whereby stressed plants actively recruit beneficial microbes via root exudates, and the complex interaction networks among microorganisms form the foundation of rhizosphere disease suppression.

We distinctly contribute the articulation of the ‘*M* genes → microbiome engineering → breeding’ axis as a central conceptual framework. This axis connects *M* genes that shape root exudate profiles to the rational design of SynComs and prebiotics, and ultimately to the breeding of crop varieties with enhanced capacity to recruit and maintain disease-suppressive microbiomes.

Strategies based on the rhizosphere microbiota, represented by SynComs and prebiotics, show great potential to replace or supplement traditional chemical control. The rapid development of omics, culturomics, and bioinformatics has greatly advanced our understanding of the structure and function of rhizosphere microbiota. Although challenges remain in field stability, mechanistic depth, and large-scale application, deep analysis of plant–microbe–environment interactions, design of smarter microbial products, integration with soil health management and crop breeding, and especially the translation of *M* genes into breeding programs will provide critical support for sustainable agriculture and green control of soil-borne diseases.

## Figures and Tables

**Table 1:** Key root exudate signals and recruited beneficial microbes.

Root Exudate Signal	Recruited Microbes	Target Disease	Crop	Reference
Malic acid	*Sphingomonas*	Bacterial wilt	Tomato	[14]
Organic acids	*Comamonadaceae*, *Xanthomonas*	*Fusarium* wilt	Cucumber	[15]
Methyl ferulate	*Bacillus*	Black shank	Tobacco	[16]
Salicylic acid	*Rhodanobacter*, *Sphingomonas*	*Fusarium* wilt	Watermelon	[17]
Flavonoids	*Microbacterium*, *Stenotrophomonas*	Clubroot	Cabbage	[18]
Taxifolin	*Bacillus* spp.	*Verticillium* wilt	Tomato	[19]
Sinapyl alcohol, 6-gingerol	*Burkholderia*	*Fusarium* wilt	Chrysanthemum	[20]

## Data Availability

The original contributions presented in this study are included in the article. Further inquiries can be directed to the corresponding authors.
